# Supplementary material for: Modeling glioblastoma heterogeneity as a dynamic network of cell states
Source: Mol Syst Biol. 2021 Sep 16;17(9):e10105. doi: 10.15252/msb.202010105 (PMC8444284; doi:10.15252/msb.202010105)
Supplement: Supplementary file 6 — Source Data for Figure 5 [file MSB-17-e10105-s004.zip › Figure5A_sourcedata/GSEA_3017/hallmarks_stateE.GseaPreranked.1621934677158/HALLMARK_E2F_TARGETS.html]

Details for gene set HALLMARK\_E2F\_TARGETS[GSEA]

|  || Dataset | state63017 |
| Phenotype | NoPhenotypeAvailable |
| Upregulated in class | na\_neg |
| GeneSet | HALLMARK\_E2F\_TARGETS |
| Enrichment Score (ES) | -0.5653252 |
| Normalized Enrichment Score (NES) | -3.0974176 |
| Nominal p-value | 0.0 |
| FDR q-value | 0.0 |
| FWER p-Value | 0.0 |
Table: GSEA Results Summary

  

Fig 1: Enrichment plot: HALLMARK\_E2F\_TARGETS      
 Profile of the Running ES Score & Positions of GeneSet Members on the Rank Ordered List

  

| PROBE | GENE SYMBOL | GENE\_TITLE | RANK IN GENE LIST | RANK METRIC SCORE | RUNNING ES | CORE ENRICHMENT || 1 | DONSON |  |  | 274 | -0.251 | -0.3293 | No |
| 2 | RAN |  |  | 298 | -0.257 | -0.3535 | No |
| 3 | HMGA1 |  |  | 313 | -0.261 | -0.3667 | No |
| 4 | EED |  |  | 316 | -0.262 | -0.3653 | No |
| 5 | PSIP1 |  |  | 319 | -0.263 | -0.3640 | No |
| 6 | TUBG1 |  |  | 323 | -0.263 | -0.3638 | No |
| 7 | RAD51C |  |  | 333 | -0.264 | -0.3709 | No |
| 8 | PAICS |  |  | 336 | -0.265 | -0.3695 | No |
| 9 | MCM5 |  |  | 337 | -0.265 | -0.3656 | No |
| 10 | STAG1 |  |  | 360 | -0.271 | -0.3884 | No |
| 11 | CDC25A |  |  | 368 | -0.273 | -0.3930 | No |
| 12 | POP7 |  |  | 370 | -0.273 | -0.3902 | No |
| 13 | SLBP |  |  | 386 | -0.277 | -0.4044 | No |
| 14 | NASP |  |  | 410 | -0.285 | -0.4282 | No |
| 15 | JPT1 |  |  | 439 | -0.296 | -0.4579 | No |
| 16 | CTCF |  |  | 443 | -0.297 | -0.4573 | No |
| 17 | HNRNPD |  |  | 456 | -0.301 | -0.4675 | No |
| 18 | MCM4 |  |  | 457 | -0.301 | -0.4631 | No |
| 19 | UBR7 |  |  | 459 | -0.302 | -0.4599 | No |
| 20 | DNMT1 |  |  | 466 | -0.304 | -0.4628 | No |
| 21 | SMC1A |  |  | 472 | -0.307 | -0.4644 | No |
| 22 | NOP56 |  |  | 483 | -0.309 | -0.4721 | No |
| 23 | NUP205 |  |  | 495 | -0.313 | -0.4809 | No |
| 24 | RFC2 |  |  | 498 | -0.313 | -0.4788 | No |
| 25 | PA2G4 |  |  | 501 | -0.314 | -0.4767 | No |
| 26 | BARD1 |  |  | 510 | -0.318 | -0.4818 | No |
| 27 | POLA2 |  |  | 521 | -0.320 | -0.4893 | No |
| 28 | MSH2 |  |  | 548 | -0.332 | -0.5161 | No |
| 29 | PLK4 |  |  | 551 | -0.334 | -0.5137 | No |
| 30 | MCM3 |  |  | 560 | -0.340 | -0.5185 | No |
| 31 | RAD1 |  |  | 564 | -0.341 | -0.5172 | No |
| 32 | TCF19 |  |  | 569 | -0.342 | -0.5170 | No |
| 33 | CCNE1 |  |  | 571 | -0.344 | -0.5133 | No |
| 34 | HMGB3 |  |  | 577 | -0.348 | -0.5143 | No |
| 35 | NUP107 |  |  | 586 | -0.352 | -0.5189 | No |
| 36 | DSCC1 |  |  | 587 | -0.352 | -0.5138 | No |
| 37 | DDX39A |  |  | 590 | -0.353 | -0.5111 | No |
| 38 | PRIM2 |  |  | 607 | -0.362 | -0.5253 | No |
| 39 | KIF18B |  |  | 612 | -0.363 | -0.5249 | No |
| 40 | MRE11 |  |  | 623 | -0.371 | -0.5317 | No |
| 41 | MTHFD2 |  |  | 625 | -0.372 | -0.5275 | No |
| 42 | EXOSC8 |  |  | 630 | -0.376 | -0.5269 | No |
| 43 | RNASEH2A |  |  | 633 | -0.378 | -0.5238 | No |
| 44 | CCP110 |  |  | 636 | -0.381 | -0.5208 | No |
| 45 | POLE |  |  | 652 | -0.395 | -0.5332 | No |
| 46 | DCK |  |  | 659 | -0.400 | -0.5347 | No |
| 47 | CIT |  |  | 680 | -0.418 | -0.5530 | No |
| 48 | RPA2 |  |  | 688 | -0.424 | -0.5553 | No |
| 49 | DIAPH3 |  |  | 696 | -0.427 | -0.5576 | No |
| 50 | RANBP1 |  |  | 697 | -0.427 | -0.5514 | No |
| 51 | CHEK1 |  |  | 704 | -0.433 | -0.5525 | No |
| 52 | TIMELESS |  |  | 707 | -0.435 | -0.5486 | No |
| 53 | POLD3 |  |  | 710 | -0.437 | -0.5447 | No |
| 54 | RAD21 |  |  | 728 | -0.458 | -0.5587 | Yes |
| 55 | SPAG5 |  |  | 729 | -0.458 | -0.5520 | Yes |
| 56 | GINS1 |  |  | 730 | -0.459 | -0.5454 | Yes |
| 57 | CSE1L |  |  | 736 | -0.461 | -0.5448 | Yes |
| 58 | LBR |  |  | 739 | -0.463 | -0.5405 | Yes |
| 59 | E2F8 |  |  | 741 | -0.467 | -0.5349 | Yes |
| 60 | SMC3 |  |  | 743 | -0.470 | -0.5293 | Yes |
| 61 | TK1 |  |  | 747 | -0.480 | -0.5260 | Yes |
| 62 | LIG1 |  |  | 752 | -0.485 | -0.5238 | Yes |
| 63 | CDCA3 |  |  | 759 | -0.497 | -0.5239 | Yes |
| 64 | TRIP13 |  |  | 760 | -0.498 | -0.5167 | Yes |
| 65 | MMS22L |  |  | 761 | -0.500 | -0.5094 | Yes |
| 66 | MXD3 |  |  | 763 | -0.502 | -0.5033 | Yes |
| 67 | GINS4 |  |  | 765 | -0.510 | -0.4972 | Yes |
| 68 | STMN1 |  |  | 778 | -0.529 | -0.5041 | Yes |
| 69 | NCAPD2 |  |  | 784 | -0.540 | -0.5023 | Yes |
| 70 | RPA3 |  |  | 788 | -0.550 | -0.4980 | Yes |
| 71 | CDC20 |  |  | 793 | -0.562 | -0.4947 | Yes |
| 72 | RACGAP1 |  |  | 796 | -0.566 | -0.4889 | Yes |
| 73 | ASF1B |  |  | 798 | -0.568 | -0.4819 | Yes |
| 74 | RFC3 |  |  | 801 | -0.569 | -0.4760 | Yes |
| 75 | TUBB |  |  | 802 | -0.570 | -0.4678 | Yes |
| 76 | USP1 |  |  | 806 | -0.575 | -0.4631 | Yes |
| 77 | CDCA8 |  |  | 808 | -0.585 | -0.4558 | Yes |
| 78 | PLK1 |  |  | 809 | -0.586 | -0.4473 | Yes |
| 79 | PSMC3IP |  |  | 811 | -0.588 | -0.4400 | Yes |
| 80 | DEK |  |  | 812 | -0.591 | -0.4314 | Yes |
| 81 | DEPDC1 |  |  | 815 | -0.596 | -0.4252 | Yes |
| 82 | MCM7 |  |  | 817 | -0.599 | -0.4177 | Yes |
| 83 | LMNB1 |  |  | 818 | -0.606 | -0.4089 | Yes |
| 84 | AURKA |  |  | 824 | -0.623 | -0.4059 | Yes |
| 85 | AURKB |  |  | 829 | -0.634 | -0.4016 | Yes |
| 86 | HELLS |  |  | 830 | -0.635 | -0.3924 | Yes |
| 87 | PCNA |  |  | 832 | -0.640 | -0.3843 | Yes |
| 88 | CDKN3 |  |  | 836 | -0.653 | -0.3785 | Yes |
| 89 | ANP32E |  |  | 841 | -0.676 | -0.3735 | Yes |
| 90 | KIF22 |  |  | 842 | -0.678 | -0.3637 | Yes |
| 91 | BIRC5 |  |  | 843 | -0.679 | -0.3538 | Yes |
| 92 | CENPM |  |  | 844 | -0.682 | -0.3439 | Yes |
| 93 | BRCA1 |  |  | 846 | -0.691 | -0.3351 | Yes |
| 94 | SPC24 |  |  | 848 | -0.694 | -0.3263 | Yes |
| 95 | BRCA2 |  |  | 850 | -0.694 | -0.3174 | Yes |
| 96 | KIF2C |  |  | 855 | -0.721 | -0.3118 | Yes |
| 97 | KIF4A |  |  | 861 | -0.736 | -0.3072 | Yes |
| 98 | HMMR |  |  | 863 | -0.740 | -0.2977 | Yes |
| 99 | CCNB2 |  |  | 865 | -0.745 | -0.2881 | Yes |
| 100 | ORC6 |  |  | 870 | -0.766 | -0.2819 | Yes |
| 101 | BUB1B |  |  | 871 | -0.766 | -0.2707 | Yes |
| 102 | MELK |  |  | 874 | -0.776 | -0.2619 | Yes |
| 103 | TMPO |  |  | 875 | -0.778 | -0.2506 | Yes |
| 104 | PTTG1 |  |  | 880 | -0.791 | -0.2440 | Yes |
| 105 | EZH2 |  |  | 886 | -0.810 | -0.2383 | Yes |
| 106 | UBE2S |  |  | 887 | -0.815 | -0.2265 | Yes |
| 107 | RAD51AP1 |  |  | 894 | -0.853 | -0.2214 | Yes |
| 108 | KPNA2 |  |  | 895 | -0.858 | -0.2089 | Yes |
| 109 | CENPE |  |  | 899 | -0.873 | -0.1999 | Yes |
| 110 | SMC4 |  |  | 902 | -0.886 | -0.1895 | Yes |
| 111 | DUT |  |  | 903 | -0.887 | -0.1766 | Yes |
| 112 | CKS1B |  |  | 909 | -0.912 | -0.1695 | Yes |
| 113 | CKS2 |  |  | 911 | -0.922 | -0.1573 | Yes |
| 114 | MYBL2 |  |  | 914 | -0.959 | -0.1458 | Yes |
| 115 | MAD2L1 |  |  | 921 | -0.990 | -0.1387 | Yes |
| 116 | UBE2T |  |  | 922 | -0.994 | -0.1243 | Yes |
| 117 | DLGAP5 |  |  | 923 | -0.998 | -0.1098 | Yes |
| 118 | SPC25 |  |  | 924 | -1.033 | -0.0948 | Yes |
| 119 | ATAD2 |  |  | 933 | -1.162 | -0.0877 | Yes |
| 120 | HMGB2 |  |  | 937 | -1.238 | -0.0734 | Yes |
| 121 | CDK1 |  |  | 938 | -1.248 | -0.0553 | Yes |
| 122 | RRM2 |  |  | 940 | -1.403 | -0.0361 | Yes |
| 123 | MKI67 |  |  | 941 | -1.407 | -0.0157 | Yes |
| 124 | TOP2A |  |  | 942 | -1.417 | 0.0049 | Yes |
Table: GSEA details [plain text format]

  

Fig 2: HALLMARK\_E2F\_TARGETS: Random ES distribution      
 Gene set null distribution of ES for **HALLMARK\_E2F\_TARGETS**
